# Can profiles of poly- and Perfluoroalkyl substances (PFASs) in human serum provide information on major exposure sources?

**DOI:** 10.1186/s12940-018-0355-4

**Published:** 2018-02-01

**Authors:** Xindi C. Hu, Clifton Dassuncao, Xianming Zhang, Philippe Grandjean, Pál Weihe, Glenys M. Webster, Flemming Nielsen, Elsie M. Sunderland

**Affiliations:** 1 Harvard T.H. Chan School of Public Health, Boston, MA 02215 USA; 2 Harvard John A. Paulson School of Engineering and Applied Sciences, Harvard University, 128 Pierce Hall, Cambridge, MA 02138 USA; 3 University of Southern Denmark, DK-5000 Odense, Denmark; 4 The Faroese Hospital System, FR-100 Tórshavn, Faroe Islands; 5 Simon Fraser University, Burnaby, BC V5A 1S6 Canada

**Keywords:** Serum, Fish and shellfish, Consumer products, Source attribution, Homologues, Perfluoroalkyl carboxylates (PFCAs)

## Abstract

**Background:**

Humans are exposed to poly- and perfluoroalkyl substances (PFASs) from diverse sources and this has been associated with negative health impacts. Advances in analytical methods have enabled routine detection of more than 15 PFASs in human sera, allowing better profiling of PFAS exposures. The composition of PFASs in human sera reflects the complexity of exposure sources but source identification can be confounded by differences in toxicokinetics affecting uptake, distribution, and elimination. Common PFASs, such as perfluorooctanoic acid (PFOA), perfluorooctane sulfonic acid (PFOS) and their precursors are ubiquitous in multiple exposure sources. However, their composition varies among sources, which may impact associated adverse health effects.

**Methods:**

We use available PFAS concentrations from several demographic groups in a North Atlantic seafood consuming population (Faroe Islands) to explore whether chemical fingerprints in human sera provide insights into predominant exposure sources. We compare serum PFAS profiles from Faroese individuals to other North American populations to investigate commonalities in potential exposure sources. We compare individuals with similar demographic and physiological characteristics and samples from the same years to reduce confounding by toxicokinetic differences and changing environmental releases.

**Results:**

Using principal components analysis (PCA) confirmed by hierarchical clustering, we assess variability in serum PFAS concentrations across three Faroese groups. The first principal component (PC)/cluster consists of C9-C12 perfluoroalkyl carboxylates (PFCAs) and is consistent with measured PFAS profiles in consumed seafood. The second PC/cluster includes perfluorohexanesulfonic acid (PFHxS) and the PFOS precursor N-ethyl perfluorooctane sulfonamidoacetate (N-EtFOSAA), which are directly used or metabolized from fluorochemicals in consumer products such as carpet and food packaging. We find that the same compounds are associated with the same exposure sources in two North American populations, suggesting generalizability of results from the Faroese population.

**Conclusions:**

We conclude that PFAS homologue profiles in serum provide valuable information on major exposure sources. It is essential to compare samples collected at similar time periods and to correct for demographic groups that are highly affected by differences in physiological processes (e.g., pregnancy). Information on PFAS homologue profiles is crucial for attributing adverse health effects to the proper mixtures or individual PFASs.

**Electronic supplementary material:**

The online version of this article (10.1186/s12940-018-0355-4) contains supplementary material, which is available to authorized users.

## Background

Poly- and perfluoroalkyl substances (PFASs) are widely used consumer and industrial chemicals that are now detectable in virtually all human populations [1–6]. Exposure to PFASs has been associated with many adverse health effects including developmental, metabolic and immune disorders, and increased risk of certain cancers [7–9]. PFAS exposure sources are diverse and include dust, food, drinking water, and many consumer products such as food packaging, outdoor gear, dental floss, carpets and furniture coatings [10–12]. Human populations are therefore exposed to different PFAS mixtures. The relative importance of different PFAS exposure sources has proven difficult to discern, both within and across populations. This information is essential for attributing adverse effects to particular PFASs or mixtures and for prioritizing actions to minimize health risks. Complex mixtures of PFASs in human sera may be further altered by variability in toxicokinetics. Improved classification of particular PFAS mixtures may allow chemical fingerprinting of PFASs in human sera and eventually lead to characterization of major causative profiles within populations and across demographic groups.

The relative importance of different PFAS exposure sources can be highly variable across populations and locations. Exposures from seafood consumption are important in many coastal communities [4, 13–15]. Other food items of animal origin have been identified as important contributors to exposure in a few other populations [16, 17]. Children and toddlers are more highly exposed to certain PFASs due to trans-placental transfer, breastfeeding, and frequent hand-to-mouth contact leading to ingestion of house dust [18–20]. Other populations may be exposed to specific PFASs from drinking water [21, 22]. Exposures estimated from measured concentrations in food, dust, and drinking water can be imprecise due to large inter-individual differences in behavior and wide ranges in environmental concentrations [23]. This imprecision is compounded when environmental measurements are not available for a specific study population and must be inferred from other studies. For example, Lorber and Egeghy [23] modeled US population exposures using PFAS concentrations in foods from another population [24] and resulting dietary perfluorooctanoic acid (PFOA) intake estimates ranged by more than one order of magnitude [23]. Others have developed multi-compartment pharmacokinetic models for individual PFASs that reproduce observed serum concentrations [25, 26]. However, such modeling approaches are typically data-intensive and focus on individual PFASs rather than chemical mixtures. Toxicokinetic data needed to parameterize such models are limited for PFASs other than PFOS, PFOA and PFHxS.

Serum concentrations of PFASs reflect accumulated exposures to multiple compounds that occurred simultaneously, providing a composite measure of uptake from all pathways and sources [23, 27]. Previous work has successfully used congener composition within chemical classes such as polychlorinated biphenyls (PCBs) to identify the origin of major human exposure sources [28–30]. Applying such techniques to PFASs is complicated by dramatic shifts in production over time and the complex metabolism of PFAS precursors. This means that differences in exposure can be inferred only from samples collected at similar time periods, and physiological differences must be carefully considered. Previously, we showed that the measured PFAS composition in surface water provides useful information on sources of environmental pollution [31]. Here, we discuss the potential feasibility of extending this approach to consider human sera and the caveats associated with such methods.

The main objective of this study is to assess exposure information that can be derived from serum-PFAS composition. We analyzed a suite of 19 PFASs in archived serum samples from past epidemiological studies in the Faroe Islands, a North Atlantic fishing community with a wide-range of PFAS exposures [14, 32]. We compare the measured composition of detectable PFASs in serum from Faroese women, children and men using multivariate statistical tools to identify clustering of major PFASs. We consider potential confounding due to inter-individual differences in toxicokinetics and elimination pathways, and test generalizability of information derived from the Faroese cohort using serum PFAS data from two other North American populations [33, 34].

## Methods

### Sample selection

We selected archived serum samples from adult men, women and children in the Faroe Islands that were available for overlapping time periods. The Faroese are a fairly homogeneous population consisting of individuals of mainly Scandinavian descent. Sera from the same children (*n* = 52) were collected at age 7 between 2004-05 and at age 13 between 2011-12 as part of a larger prospective birth cohort described elsewhere [32]. Adult male serum samples (*n* = 10) were collected in 2006 from individuals who reported participating in pilot whale harvesting (whalers) and were thus expected to have the highest PFAS exposures from seafood [14]. Women (*n* = 52) were part of a different prospective cohort established in 2007. Blood samples were collected 2 weeks after singleton delivery in 2007–08 [35, 36]. For children, the 2004–05 serum collection was compared with Faroese whalers and women from the overlapping time periods, while the 2011–12 serum collection was compared to North American children of the same age from the same time period, as further described below.

Dietary questionnaire data on seafood consumption, such as the number of whale meat meals per month, were available for all Faroese individuals. Imprecision in dietary survey data is well established due to several types of recall bias [37]. Measured hair mercury concentrations provide an alternative empirical proxy for seafood exposure because methylmercury is almost exclusively from fish, shellfish and marine mammal consumption [37]. Thus, we use previously reported hair mercury data from Faroese subjects as an additional indicator for seafood consumption [38]. All mothers and other participants provided written informed consent, and study protocols were reviewed and approved by the Faroese ethical review committee and the Institutional Review Board of Harvard T.H. Chan School of Public Health.

### Sample analysis for PFASs

Blood samples (10 mL) from Faroese individuals were allowed to clot for approximately 30 min and spun at 2000 g for 10 min to separate the serum. Serum samples were transferred into polypropylene cryogenic storage vials (2.5 or 5 mL) and stored at − 80 °C until analysis. PFASs (Table 1) were analyzed by online solid-phase extraction and high-pressure liquid chromatography with tandem mass spectrometry (HPLC-MS/MS) [39]. For each sample, 150 μL serum was pipetted into a 2 mL centrifuge tube. Internal standard solution (30 μL, 50 ng mL^− 1^ for perfluorooctane sulfonic acid (PFOS) and 20 ng mL^− 1^ for other PFASs, Wellington Laboratories, Canada), and methanol (120 μL) were added prior to mixing on a whirl mixer. Calibration solutions were prepared in serum from newborn calves (Biological Industries, Israel) because it matches the matrix of human serum and contains low levels of PFASs. Solutions spanned a concentration range from 0.050 to 100 ng PFASs mL^− 1^ serum for all analytes. Both calibration solutions and samples were whirl mixed and centrifuged at 15,000 rpm for 20 min. 160 μL of the supernatant was transferred to a polypropylene autosampler vial. Formic acid (400 μL, 0.1 M) was added and the solution was mixed on a whirl mixer and placed in the autosampler for injection into HPLC-MS/MS system. A volume of 400 μL was injected into the on-line solid-phase extraction column.

**Table 1:** List of quantified PFASs in Faroese serum samples and their limits of detection

Analyte	Acronym	Carbon-chain length	Molecular ion	LOD (ng/mL)
*Carboxylic acids (PFCAs)*				
Perfluorobutanoic acid	PFBA	C-4	F(CF_2_)_3_CO_2_^−^	0.1
Perfluoropentanoic acid	PFPeA	C-5	F(CF_2_)_4_CO_2_^−^	0.05
Perfluorohexanoic acid	PFHxA	C-6	F(CF_2_)_5_CO_2_^−^	0.05
Perfluoroheptanoic acid	PFHpA	C-7	F(CF_2_)_6_CO_2_^−^	0.03
Perfluorooctanoic acid	PFOA	C-8	F(CF_2_)_7_CO_2_^−^	0.03
Perfluorononanoic acid	PFNA	C-9	F(CF_2_)_8_CO_2_^−^	0.03
Perfluorodecanoic acid	PFDA	C-10	F(CF_2_)_9_CO_2_^−^	0.03
Perfluoroundecanoic acid	PFUnDA	C-11	F(CF_2_)_10_CO_2_^−^	0.03
Perfluorododecanoic acid	PFDoDA	C-12	F(CF_2_)_11_CO_2_^−^	0.05
*Sulfonic acids (PFSAs)*				
Perfluorobutane sulfonic acid	PFBS	C-4	F(CF_2_)_4_SO_3_^−^	0.1
Perfluorohexane sulfonic acid	PFHxS	C-6	F(CF_2_)_6_SO_3_^−^	0.05
Perfluoroheptane sulfonic acid	PFHpS	C-7	F(CF_2_)_7_SO_3_^−^	0.03
Linear perfluorooctane sulfonic acid	nPFOS	C-8	F(CF_2_)_8_SO_3_^−^	0.03
Branched perfluorooctane sulfonic acid	brPFOS	C-8	F(CF_2_)_8_SO_3_^−^	0.03
Perfluorodecane sulfonic acid	PFDS	C-10	F(CF_2_)_10_SO_3_^−^	0.03
*PFOS Precursors (PreFOS)*				
N-ethyl perfluorooctane sulfonamidoacetate	N-EtFOSAA	C-8	F(CF_2_)_8_SO_2_N (C_2_H_5_)CH_2_CO_2_^−^	0.03
N-methyl perfluorooctane sulfonamidoacetate	N-MeFOSAA	C-8	F(CF_2_)_8_SO_2_N (CH_3_)CH_2_CO_2_^−^	0.03
Linear perfluorooctane sulfonamide	nFOSA	C-8	F(CF_2_)_8_SO_2_NH_2_	0.03
Branched perfluorooctane sulfonamide	brFOSA	C-8	F(CF_2_)_8_SO_2_NH_2_	0.03

In each analytical series (~ 50 samples), quality control serum samples, calibration standards, and reagent and serum blanks were included to ensure the accuracy and reliability of data. Within-batch and between-batch coefficient of variations were lower than 8.9% and 12.9% for all analytes. All serum samples were analyzed for 19 PFASs and the limit of detection (LOD) for PFASs was 0.03–0.1 ng mL^− 1^ (Table 1). Four analytes (PFBA, PFPeA, PFHxA and PFBS) were below the LOD for all Faroese children and women, and were therefore excluded as target analytes for the Faroese whaling men.

### Other study populations

We compared serum PFAS data from the Faroese to exposure information from two North American cohorts studied at overlapping time periods. Most consumer products that are potential sources of PFAS exposure in the Faroe Islands are imported. Thus, use of consumer products in the Faroes is expected to result in similar exposures to PFASs as in European and North American populations. PFASs with longer carbon chain length are well established to have greater propensity to bioaccumulate in freshwater and marine fish, shellfish and marine mammals [40, 41], providing a characteristic exposure signature for seafood consumers across many populations [42–44].

Our analysis used serum PFAS data from the US National Health and Nutrition Examination Survey (NHANES), a cross-sectional survey of the U.S general population [34]. Questionnaire data and serum PFAS levels for NHANES are available for *n* = 2120 individuals in 2005–06 (age > 12 years, 50.6% female) and for *n* = 1751 individuals in 2011–12 (age > 12 years, 48.7% female). We downloaded serum PFAS and seafood consumption data, along with other relevant predictors, such as data on demographic characteristics and reproductive health, and merged them into one file [34]. Laboratory methods are available on the Center for Disease Control and Prevention (CDC) website and the LOD for different PFASs ranged between 0.1–0.4 ng mL^− 1^ [45].

We also synthesized exposure information and behavior data from the Chemicals, Health and Pregnancy (CHirP) cohort established in Vancouver, Canada [46, 47]. Between December 2006 and June 2008, the CHirP study enrolled and collected blood samples from 152 pregnant women in their first trimester. Data on potential covariates such as demographic attributes, dietary habits, and use of consumer products were collected using online and in-person questionnaires between weeks 19 to 24 of the pregnancy. Serum samples were analyzed for 23 PFASs at ALS Laboratory in Edmonton, Canada by HPLC-MS/MS following a solid-phase extraction method similar to Kuklenyik et al. [46, 48]. The LOD in CHirP was higher than the Faroese and NHANES studies (0.5 ng mL ^− 1^ for all PFASs in serum) and only four PFASs (PFOS, PFOA, PFNA and PFHxS) were detectable in more than 50% of participants. Participant characteristics of NHANES 2005–06 and CHirP are outlined in Additional file 1: Table S1 and Table S2.

### Statistical methods

Multiple statistical techniques were used to handle the left censored data on serum PFAS concentrations. For the main analysis, we used simple substitution by the LOD multiplied by 1/√2. We then tested the impacts of this assumption using the robust regression on order statistics (ROS) method to impute the missing data containing multiple detection limits [49, 50]. All 15 PFASs were frequently detected among Faroese whaling men (60% - 100%). We included six compounds with low frequency of detection in Faroese children (12% - 100%) and women (2% - 27%) because variability in their detection provides useful information on potential exposure sources.

For each PFAS, we used analysis of variance (ANOVA) to compare mean serum concentrations among Faroese whaling men, children, and women. Significant differences were identified using Tukey’s honestly significant difference (HSD) tests with *α* = 0.05 (R package *agricolae*) [51]. For Faroese children, we calculated Spearman correlation coefficients between serum PFAS concentrations and factors reflecting seafood consumption (hair mercury and whale consumption frequency).

We used principal component analysis (PCA) and hierarchical clustering to identify variability in PFAS profiles across Faroese men, women and children. PCA was applied to the covariance matrix of the PFAS concentration profiles (R package *FactoMineR*) [52], and the number of principal components was based on those with an eigenvalue greater than one. Hierarchical clustering with Ward’s method was used to investigate similarities among the serum PFAS profiles, defined as the squared Euclidean distance (R package *stats*) [53].

We matched the Faroese whaling men and women with individuals of similar demographic characteristics (age, gender, race) and physiological characteristics (postpartum) from NHANES 2005–06 who provided serum samples at approximately the same time period as the Faroese (Table 2). Selection of the same collection period, and similar demographic and physiological characteristics in these cohorts reduces confounding by changing production and environmental releases and toxicokinetic differences across demographic groups. A comparison with the data from Faroese children at age 7 was not possible because NHANES participants are 12 years and older. We thus used data from the same Faroese cohort in 2011–12 when they reached age 13 to compare to age matched US individuals from NHANES 2011–12. Related work suggests gender differences among young teens does not influence PFAS body burdens [54].

**Table 2:** Characteristics of Faroese and NHANES individuals included in the PCA analysis

	Men		Children		Women^*^	
	Faroese	NHANES	Faroese	NHANES	Faroese	NHANES
*n*	10	56	51	30	51	23
Sampling year	2006	2005–2006	2011–2012	2011–2012	2007–2008	2005–2006
Age	47–80	47–80	13	12–14	19–44	19–44
Race/ethnicity	White	White	White	White	White	White

^*^Note: Faroese women were two weeks postpartum and NHANES women were within one year postpartum

We selected 56 white men and 23 white women within one year-postpartum period from NHANES 2005–06 and 30 white children from NHANES 2011–12. We projected these NHANES population onto the principal components derived from PCA analysis on the Faroese, and assign scores based on the original loadings. Unlike the Faroese serum PFASs measurements, NHANES data does not distinguish between linear and branched PFOS, and linear and branched FOSA. To compare to Faroese measurements, we thus assumed NHANES data contained 69% linear and 31% branched based on literature values for Norwegian adults [55].

We developed multivariable regression models for the NHANES 2005–06 (*n* = 2120) and CHirP cohorts (*n* = 151) based on measured PFAS levels in serum and different potential drivers of PFAS exposures such as seafood consumption, consumer product use and drinking water sources. We focused on compounds identified in the PCA unless the detection frequency was < 50%. The multivariable regression model was used to predict serum levels of individual PFASs and thus differs from the PCA that selected for co-varying compounds. In NHANES, seafood consumption was derived from a 30-day food frequency questionnaire.

Information on consumer product use is limited in NHANES. A study of household dust in NHANES 2005–06 includes data on floor cover, allowing us to infer the use of carpet cleaning products. Other variables include age, gender, race, household income, health factors such as menstrual status, and potential exposure sources such as tap water sources, past military service and country of birth [56].

The CHirP study collected detailed information on seafood consumption, and consumer product use including food packaging (popcorn bags, takeout and paper cups), carpet cleaner, car polishes, non-stick cookware, waterproof sprays and waxes. Frequencies were recorded in either categorical format such as “have used carpet repellent or not in the past 3 years,” or continuous format such as “number of takeout meals per year in the year before pregnancy.” We selected the best multivariable model of PFAS serum concentrations in the CHirP cohort using a supervised forward stepwise procedure.

We first tested linear and nonlinear effects of each independent variable on the log-transformed serum PFAS levels using the generalized additive models (GAMs, R package *gam*) with a cubic spline smoothing function [57]. Predictors with *p*-values below 0.05 were retained for inclusion in the subsequent multivariable models. We present results in tabular format for parametric terms, and in graphic format for smoothed terms. For regression results, we calculate percent difference in PFAS concentrations associated with each predictor by exponentiating regression coefficients, minus one, and multiplying by 100%.

## Results

### Serum PFAS profiles among Faroese individuals

**Fig. 1:**
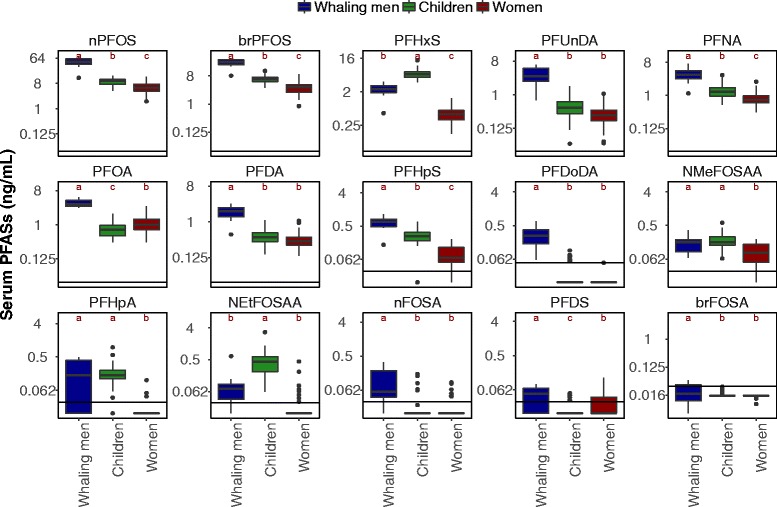
Measured PFAS concentrations in sera from Faroese individuals collected between 2004 and 2007. Horizontal lines on the boxplot represent the 25th, 50th and 75th percentiles, and whiskers extend to the highest and lowest value within 1.5 times interquartile range. Different letters above box-and-whisker plots indicate significant differences among Faroese demographic groups (Tukey HSD test, *α* = 0.05). Black horizontal lines indicate level of detection (LOD) for each compound. Concentrations below LOD were included as LOD multiplied by 1/√2

Serum PFAS profiles for Faroese individuals shown in Fig. 1 reflect combined exposures from different sources and toxicokinetic processes affecting elimination. The sum of PFASs measured here are statistically greater in whaling men, followed by children, and lowest in women (Fig. 1, one-way ANOVA, *p* < 0.001). Sensitivity analyses using the ROS method to impute censored data generated similar results (Fig. S1). PFAS profiles of whaling men are dominated by PFOS, which accounts for almost 80% of the sum of PFASs (Fig. S2). Faroese children at age 7 have significantly elevated concentrations of PFHxS and N-EtFOSAA, compared to whaling men and women (Tukey HSD test, *α* = 0.05, Table 3, Fig. 1).

**Table 3:** PFAS concentrations (ng mL^−1^) measured in serum samples collected from Faroese whaling men (M, *n* = 10), children (C, *n* = 51), and women (W, *n* = 51) between 2004-07

Analyte	Detection Frequency (%)			Median			Range		
	M	C	W	M	C	W	M	C	W
nPFOS	100	100	100	47.8	9.82	5.59	12.66–76.04	4.40–15.0	1.84–13.8
brPFOS	100	100	100	22.4	6.38	3.43	8.13–28.83	3.39–11.6	0.86–9.08
PFHxS	100	100	100	2.38	5.94	0.5	0.53–3.71	3.59–14.3	0.15–1.35
PFUnDA	100	100	100	3.13	0.45	0.28	0.71–6.51	0.05–1.7	0.05–1.06
PFNA	100	100	100	3.76	1.24	0.76	1.14–7.39	0.56–3.58	0.35–2.37
PFOA	100	100	100	3.46	0.73	1.0	2.79–5.36	0.34–1.97	0.35–3.1
PFDA	100	100	100	1.85	0.41	0.32	0.48–2.91	0.15–1.1	0.14–1.06
PFHpS	100	90	86	0.65	0.28	0.09	0.16–1.06	<LOD - 0.65	<LOD - 0.22
PFDoDA	100	20	2	0.27	0.07	0.05	0.06–0.65	<LOD - 0.11	<LOD - 0.05
N-MeFOSAA	100	100	92	0.18	0.18	0.1	0.07–0.38	0.06–0.59	<LOD - 0.21
PFHpA	60	90	6	0.39	0.18	0.05	<LOD - 0.49	<LOD - 0.92	<LOD - 0.12
N-EtFOSAA	90	100	18	0.08	0.43	0.07	<LOD - 0.63	0.06–3.05	<LOD - 0.45
nFOSA	80	14	14	0.08	0.07	0.05	<LOD - 0.34	<LOD - 0.17	<LOD - 0.1
PFDS	60	12	27	0.07	0.04	0.06	<LOD - 0.09	<LOD - 0.05	<LOD - 0.13
brFOSA	90	14	12	0.02	0.02	0.01	<LOD - 0.05	<LOD - 0.03	<LOD - 0.01
15 PFASs				87.8	26.2	12.9	26.9–129.1	16.3–39.4	5.01–25.9

**Fig. 2:**
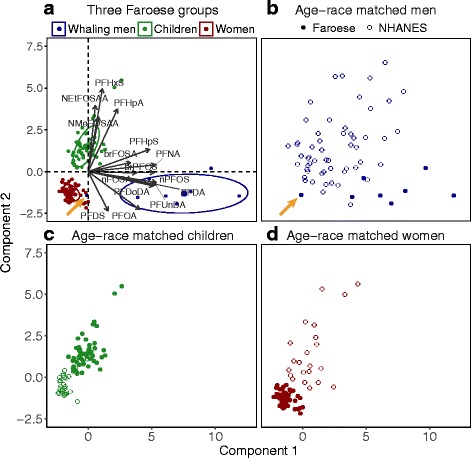
Principal component analysis (PCA) of serum PFAS profiles for (**a**) Faroese whaling men, children and women, (**b**) Faroese whaling men and US men, (**c**) Faroese children and US children, (**d**) Faroese women and US women. In Panel (**a**) and (**b**), the orange arrow points to a whaling man who did not consume pilot whale meat in the year preceding serum collection. In Panel (**b**) – (**d**), the coordinates of each data point are computed as the raw data on serum PFASs weighted by the factor loadings derived from Panel (**a**)

The first three components of the PCA explain 77% of the total variability in serum PFAS concentrations (Fig. 2a and Additional file 1 Figure S3; detailed loadings are provided in Additional file 1: Table S3). The three Faroese demographic groups have similar principal component scores within groups. Repeating the PCA analyses using the ROS method to impute censored data generated similar results (Additional file 1: Figure S4).

The first PCA component explains 51% of the variability, and clearly separates whaling men from children and women. PFOS and C9-C12 PFCAs have high loadings on the first component and are distinct from the other PFASs. The first component includes compounds with both low and high detection frequency indicating this grouping is unlikely to be driven by detection rates.

The second component explains 17% of the variability in serum PFAS profiles and has high scores among Faroese children. PFHxS, PFHpA and two PFOS precursors, N-MeFOSAA and N-EtFOSAA have high loadings in this component of the PCA. The third component explains 9% of the variability. Two PFOS precursors, nFOSA and brFOSA have high loadings in this component, but the three Faroese groups have similar scores on this component.

We compared PCA results for Faroese groups to their US counterparts with similar demographic and physiological characteristics with serum samples collected at the same time period to reduce temporal variability in the exposure signal and toxicokinetic differences (Fig. 2b–d). Faroese whaling men have higher scores on Component 1 when compared to white men from NHANES 2005–06 (Fig. 2b). Faroese children at age 13 (2011–12) have higher scores on Component 1 and Component 2 compared to age 13 white children in NHANES for the same year (Fig. 2c). Figure 2d shows the PCA results for mothers from the Faroe Islands and US white women, who are 19 to 44 years old and within the one year-postpartum period, from NHANES 2005–06. The mothers from the Faroes have lower scores on Component 1 and Component 2 compared to women in NHANES.

**Fig. 3:**
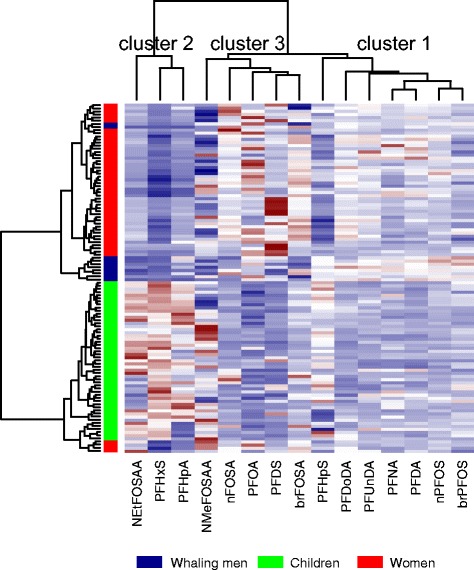
Hierarchical clustering of serum profiles measured in Faroese whaling men, children and women. Each row represents an individual and each column represents a PFAS. Red (high) and blue (low) shades represent compositions of each PFAS in each individual relative to other individuals

Similar to the PCA, results of hierarchical clustering clearly separate whaling men, children and women into distinct groups (Fig. 3). One whaling man who reported not eating any whale meat in the year prior to blood sample collection fell into the same cluster as women. Clustering of different PFASs is mostly consistent with PCA results. The cluster associated with Faroese children (cluster 2) does not include N-MeFOSAA, which is instead associated with women (cluster 3). The composition of PFASs clustered with whaling men (cluster 1) does not include PFOA and PFDS, which are again associated with women. These differences likely occur because hierarchical clustering considers all available data, whereas the first three principal components of PCA only explain 77% of the variability in PFAS composition.

**Fig. 4:**
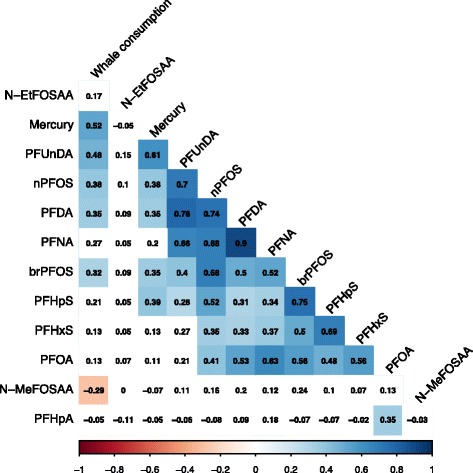
Spearman correlation matrix between hair-mercury concentrations as an indicator for seafood consumption, dietary recall data on whale consumption, and PFAS concentrations in serum from Faroese children. Statistically significant correlation coefficients are indicated by blue (positive) and red (negative) shades (*p* < 0.05). Color intensities correspond to the strength of the association

Figure 4 shows correlations between concentrations of PFASs measured in Faroese children and number of whale meat meals per month. Hair mercury concentrations, a proxy for magnitudes of seafood consumption, are significantly correlated with PFHpS, PFDA, PFOS and PFUnDA in the Faroese children (Spearman rho value: 0.35–0.61, Fig. 4). Correlations between reported whale meat consumption frequency and long-chain PFASs (such as PFDA, PFOS, and PFUnDA) are similar but slightly weaker than those observed for hair mercury and the same PFASs. This likely reflects recall bias associated with dietary survey data rather than a true difference [37].

### Predictors of PFASs in two north American populations

**Fig. 5:**
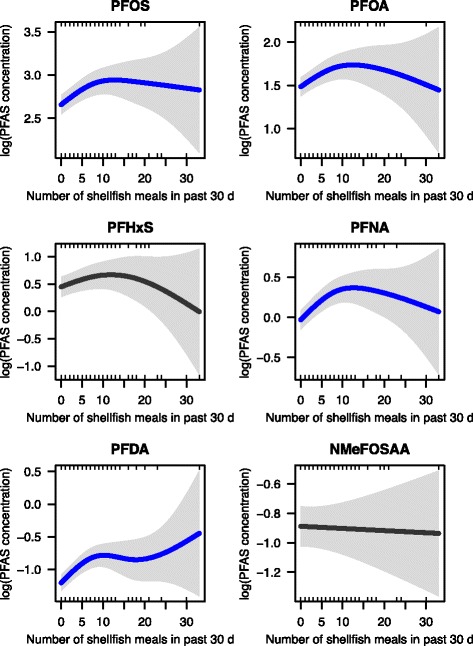
Associations between number of shellfish meals consumed in the past 30 days and serum PFAS levels in NHANES 2005–06 participants. The generalized additive model plots are controlled for age, sex, race/ethnicity, household income, body mass index (BMI), born place, prior pregnancy and breastfeeding, menstrual status, military service, tap water sources, and floor type. The solid line is the estimate and statistical significance is shown by color (blue: *p* < 0.05). Shaded grey areas represent the 95% confidence interval

We found significant associations between shellfish consumption and serum levels of PFOS, PFOA, PFNA and PFDA, after adjusting for socio-demographic factors (Additional file 1: Table S5) and other exposure sources including floor type, drinking water sources, fire-fighting foam exposure (generalized additive model, *p* < 0.05, Fig. 5). Fully or partially carpet covered floors were associated with 37.2% increase in serum PFHxS concentrations, 17.2% increase in N-MeFOSAA, and 12.2% increases in PFOS (*p* < 0.05, Additional file 1 Table S5). The final multivariable model explains 9–25% of the variance in PFAS concentrations for the six PFASs considered in the NHANES 2005–06 serum data examined here (Additional file 1 Table S5 and Fig. S5).

**Fig. 6:**
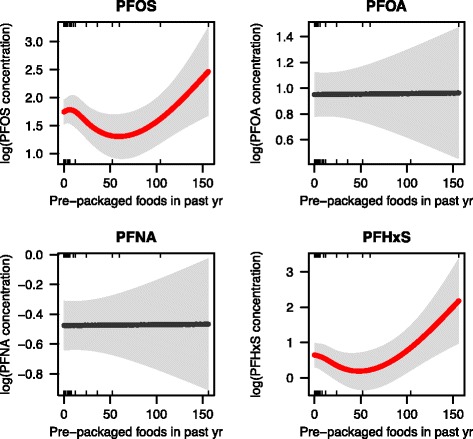
Associations between number of preheated packaged food consumed in the previous year and serum PFAS levels in CHirP participants. The generalized additive model plots are controlled for age, race/ethnicity, prior pregnancy and breastfeeding, seafood consumption and other consumer product use including carpet cleaner, fire-fighting foam, wax, non-stick cookware, etc. The solid line is the estimate and statistical significance is shown by color (red: *p* < 0.05). Shaded grey areas represent the 95% confidence interval

Unadjusted associations of potential predictors with serum PFAS concentrations measured in the CHirP study are shown in Additional file 1: Table S4. The final model explains 34–58% of the variance in PFAS concentrations for the four PFASs considered in CHirP (Additional file 1: Table S6 and Figure S6). Similar to NHANES, we found statistically significantly positive associations between shellfish consumption and increased PFNA concentrations in CHirP, but not other PFASs. We found positive associations between serum PFHxS composition and increased use of stovetop Teflon cookware and preheated packaged foods (Fig. 6). PFOS was also associated with increased use of packaged foods. These results were adjusted for PFAS exposure sources from other consumer goods, seafood consumption and demographic factors that could affect toxicokinetics such as race/ethnicity, previous pregnancy and breastfeeding history (Additional file 1: Table S6).

## Discussion

Our results link the chemical fingerprint of 15 PFASs in human serum with predominant exposure sources using measurements collected at similar times for three demographic groups in the Faroe Islands. Our analysis suggests that individuals exposed to PFASs from marine food are identifiable by an elevated proportion of C9-C12 PFCAs in their serum. Individuals who have exposures dominated by consumer products have relatively higher proportions of PFHxS, and N-EtFOSAA. We find the same chemical fingerprints are associated with the same exposure sources in the NHANES and CHirP cohorts, suggesting generalizability of results from the Faroese population.

C9-C12 PFCAs are known to bioaccumulate in aquatic food webs [41, 43, 58]. Pilot whale meat has similar composition to other seafood [40, 41, 43]. One exception is a relatively higher composition of FOSA in whale meat [42]. However, FOSA is biotransformed in humans, resulting in elevated exposures to PFOS, similar to other seafood [14, 24, 59]. This is consistent with our results (first PCA component) suggesting that C9-C12 PFCAs are effective tracers of seafood consumption. The first PCA component separates whaling men from the other Faroese groups known to consume less whale meat. Some of the compounds in this cluster (PFOS and PFOA) are commonly found in a multitude of exposure sources and thus do not serve as effective tracers. This is reflected by differences with hierarchical clustering results (Fig. 3).

Strong positive correlations between PFHpS, PFDA, PFOS, PFUnDA and hair mercury concentrations among Faroese children reinforce seafood as a common exposure source. The poor correlation between hair mercury and several other PFASs, such as PFHxS, N-MeFOSAA and N-EtFOSAA, suggests that seafood consumption patterns do not drive serum concentrations of these compounds. These results are consistent with findings from PCA and hierarchical clustering results.

In both NHANES 2005–06 and the CHirP cohort, PFNA was associated with increased seafood consumption. PFOS and PFOA are ubiquitously found in multiple exposure sources and are thus not robust tracers for any specific source. PFDA and PFUnDA were not detectable in more than 50% of study population in CHirP, therefore we did not investigate their association with shellfish consumption. Our results are consistent with Falandysz et al. [59] who first reported elevated PFAS exposures among seafood consumers on the Baltic Coast. Similarly, Haug et al. [13] estimated that seafood accounts for 93% of daily PFUnDA intake and 81% of PFOS for adults in Norway. Positive associations between seafood consumption and serum PFDA, PFNA, PFOS and PFUnDA concentrations have also been reported for NHANES data from recent years (2007–2014) [56].

The second PCA component in Fig. 2a consists of PFHxS, PFHpA and two PFOS precursors, N-MeFOSAA and N-EtFOSAA. It separates Faroese children from the other Faroese groups. N-MeFOSAA belongs to a different cluster in hierarchical clustering and is thus less likely to be a robust tracer (Fig. 3). Children have higher PFASs body burdens due to trans-placental transfer, breastfeeding, and frequent hand-to-mouth exposure [20, 26, 60]. Trans-placental transfer and breastfeeding are unlikely to fully account for the high scores on the second component for children in our study. First, the age of children (7 yrs) in this study suggests the influence of trans-placental transfer and breastfeeding on their PFAS body burdens should be small compared to other childhood exposure sources, despite the long half-lives of PFHxS (mean 7.3–8.3 years) [61–63]. Second, PFHxS and N-EtFOSAA are not the main compounds known to be transferred in breast milk. Mogensen et al. [20] observed that each month of exclusive breastfeeding was associated with a 30% increase in serum PFOS, PFOA, PFNA, and PFDA in Faroese children. In the C8 Science Panel Study, Mondal et al. [64] reported a monotonic decline in maternal serum concentrations as the duration of breastfeeding increased for PFOS, PFOA and PFNA, but not for PFHxS. Therefore, we infer that elevated exposures to PFHxS and other compounds in the second component in Faroese children reflect other postnatal sources.

Several prior surveys have identified PFHxS as one of the most abundant PFASs present in indoor dust samples [65, 66]. N-MeFOSAA and N-EtFOSAA have been detected in human serum following exposure to the volatile precursors N-methyl perfluorooctane sulfonamidoethanol (N-MeFOSE) and N-ethyl perfluorooctane sulfonamidoethanol (N-EtFOSE) [67, 68]. High levels (> 1000 ppm) of N-MeFOSE and PFHxS were reported in several 3M Scotchguard formulations [69]. N-EtFOSE was mainly used in food packaging during the time period of sample collection in this study and was detected in indoor air samples [18]. PFHxS was systematically underestimated in prior studies that modeled PFAS exposures in children through placental transfer and breastfeeding, suggesting the importance of missing postnatal exposure sources such as household dust [26]. We thus propose that N-EtFOSAA and PFHxS are both tracers of exposures that originate from consumer products.

Recent studies have reported a high prevalence of fluorinated chemicals in food packaging material [70], although potential contributions to serum-PFAS concentrations have not been quantified. Positive associations between N-EtFOSAA and PFHxS and use of carpet, non-stick cookware and food packaging were apparent in analyses using NHANES 2005–06 serum data and the CHirP cohort. Positive associations between serum PFOS concentrations and carpet use were observed in NHANES 2005–06 and between serum PFOS and use of packaged foods in the CHirP study. Migration of N-EtFOSE based chemicals from carpet cleaning products and food packaging materials and subsequent transformation to PFOS may explain these results [10, 11].

Household dust from the Faroe Islands has similar PFAS profiles to samples from Canada, Spain, Australia [71]. Our findings are consistent with other environmental monitoring studies that have linked higher PFHxS levels in indoor dust and FOSAs/FOSEs levels in indoor air to carpet area [66, 68, 71]. A recent study of Boston area children found elevated PFOS, PFHxS and N-MeFOSAA in sera from individuals who sleep in a room with carpet or a rug [60]. Other studies have found positive associations between PFOS and PFHxS and use of stain-repellant clothing, firefighting foams and consumption of microwavable foods and negative associations between PFHxS and vacuuming frequency [72, 73]. Data linking consumer product use to serum PFAS levels are scarce due to challenges associated with quantifying long-term user behavior.

By comparing Faroese groups with NHANES individuals of the same gender/race and similar age collected around the same time periods, our analysis reduces variability in serum PFAS profiles due temporal and toxicokinetic differences (Fig. 2b-d). Faroese men and children have higher scores on the PCA Component 1, which mainly consist of C9-C12 PFCAs, compared to their NHANES counterpart. This is consistent with frequent seafood consumption in the Faroes and our hypothesis that these PFASs can be tracers for exposure from seafood consumption. Statistical clusters for the two populations of 13-year old children (Fig. 2c) overlap more than the whaling men and NHANES men (Fig. 2b), suggesting greater similarity in exposure sources. These results suggest greater relative exposure from consumer products in children from the Faroe Islands compared to whaling men.

Serum samples from Faroese mothers were obtained two weeks after childbirth. Previous studies have found parous women have lower PFASs compared to nulliparous women, and PFASs levels increase with the time interval since the last pregnancy and breastfeeding period [74, 75]. We compared Faroese mothers to NHANES women who were within one year of postpartum because finer resolution data on time since delivery is not available in NHANES. The salient differences in PFASs profiles may thus reflect differences in the postpartum period for Faroese mothers (two weeks) compared to NHANES women.

## Conclusions

Recent advances in analytical chemistry have allowed numerous PFASs to be reliably detected in small volumes of human serum [39]. The suite of 15 PFASs reported in this study goes beyond standard epidemiological studies that typically report five or fewer compounds. Including additional PFASs and metabolites of precursors would increase the probability of identifying unique biomarkers for human exposure pathways [76–78]. Statistical methods presented in this paper are useful for qualitative differentiation of dominant exposure sources. This method could be enhanced by using factor analysis and receptor models to quantify the relative contribution of different sources but requires a metric for total PFAS exposure [79]. Routine measurements of extractable organic fluorine in human sera might thus complement data on individual PFASs and enrich the exposure information derived from serum samples [80–82].

Recent epidemiological research on associations between PFAS exposures and adverse human health effects has emphasized correlations with single compounds. Due to inter-correlations between PFASs, adjusted regression analyses are often non-informative. One approach for accounting for complex exposures is to apply structural equation modeling. In this approach, individual serum-PFAS concentrations contribute to a latent PFAS variable, with different loading factors. Our results show that this approach is useful but can be improved by a weighting of PFASs that reflects exposure sources. PFASs serum profiles will inform a better understanding of the health impact of PFASs with different origins. The present study illustrates that this approach is feasible and that addition of a few more PFASs than those routinely measured will make this possible.

Our analyses suggest that the composition of PFASs in human sera contains important information on dominant PFASs exposure sources that can be leveraged from samples that may be routinely collected during epidemiological studies. Individuals exposed to PFASs from seafood are identifiable by an elevated proportion of C9-C12 PFCAs in their serum, while those exposed to consumer products have relatively higher proportions of PFHxS and N-EtFOSAA. Some PFASs such as PFOS and PFOA are more ubiquitous in many exposure sources and thus do not serve as effective tracers. Routine reporting of more than the five or six PFASs that are now standard for epidemiological studies would be useful for making such inferences. Confounding factors such as temporal trends and toxicokinetic processes must be carefully considered before making inferences about differences in exposure sources.

## Additional file

Additional file 1: Supplementary figures, tables and information. (PDF 1110 kb)

## Abbreviations

ANOVA: Analysis of variance
CDC: Center for Disease Control and Prevention
CHirP: Chemicals, Health and Pregnancy
FOSA: Perfluorooctane sulfonamide
GAM: Generalized additive model
HPLC-MS/MS: High-pressure liquid chromatography with tandem mass spectrometry
HSD: Honestly significant difference
LOD: Limit of detection
N-EtFOSAA: N-ethyl perfluorooctane sulfonamidoacetate
N-EtFOSE: N-ethyl perfluorooctane sulfonamidoethanol
NHANES: National Health and Nutrition Examination Survey
N-MeFOSAA: N-methyl perfluorooctane sulfonamidoacetate
N-MeFOSE: N-methyl perfluorooctane sulfonamidoethanol
PCA: Principal components analysis
PFAA: Perfluoroalkyl acid
PFASs: Poly- and perfluoroalkyl substances
PFBA: Perfluorobutanoic acid
PFBS: Perfluorobutanesulfonic acid
PFCAs: Perfluoroalkyl carboxylates
PFDA: Perfluorodecanoic acid
PFDoDA: Perfluorododecanoic acid
PFDS: Perfluorodecanesulfonic acid
PFHpA: Perfluoroheptanoic acid
PFHpS: Perfluoroheptanesulfonic acid
PFHxA: Perfluorohexanoic acid
PFHxS: Perfluorohexanesulfonic acid
PFNA: Perfluorononanoic acid
PFOA: Perfluorooctanoic acid
PFOS: Perfluorooctanesulfonic acid
PFPeA: Perfluoropentanoic acid
PFUnDA: Perfluoroundecanoic acid
ROS: Robust regression on order statistics
