# Supplementary material for: Can profiles of poly- and Perfluoroalkyl substances (PFASs) in human serum provide information on major exposure sources?
Source: Environ Health. 2018 Feb 1;17:11. doi: 10.1186/s12940-018-0355-4 (PMC5796515; doi:10.1186/s12940-018-0355-4)
Supplement: Additional file 1: — Supplementary figures, tables and information. (PDF 1110 kb) [file 12940_2018_355_MOESM1_ESM.pdf]

Additional file 1: Supplementary figures, tables and information

Can Profiles of Poly- and Perfluoroalkyl Substances (PFASs) in Human Serum Provide Information on Major Exposure Sources?

Xindi C. Hu[a,b,*], Clifton Dassuncao[a,b], Xianming Zhang[b], Philippe Grandjean[a,c], Flemming Nielsen[c], Pal Weihe[d], Glenys M. Webster[e], Elsie M. Sunderland[a,b]

[a] Harvard T.H. Chan School of Public Health, Boston, Massachusetts 02215, United States

[b] Harvard John A. Paulson School of Engineering and Applied Sciences, Cambridge, Massachusetts 02138, United States

[c] University of Southern Denmark, DK-5000 Odense, Denmark

[d] The Faroese Hospital System, FR-100 Tórshavn, Faroe Islands

[e] Simon Fraser University, Burnaby, British Columbia V5A 1S6, Canada

Corresponding Author:

*Phone: 1-617-496-4653. E-mail: xhu@mail.harvard.edu. Mail: 128 Pierce Hall, Harvard University, Cambridge, Massachusetts, United States 02138.

Contents of this File:

Contents	Page
Table S1. Characteristics of NHANES 2005-06 participants included in GAM analysis (unweighted, *n*=2,120)	3
Table S2. Characteristics of CHirP participants included in GAM analysis (*n*=152)	4
Table S3. Loadings of the first three principal components (PCs) after varimax rotation.	5
Table S4. Screening predictors for PFASs in CHirP using univariate regression	6
Table S5 Associations between exposure behavioral factors and serum PFAS levels in NHANES 2005-06 population (parametric terms only)	8
Table S6 Associations between exposure behavioral factors and serum PFAS levels in the CHirP cohort (parametric terms only)	10
Fig. S1. Sensitivity analysis for mean serum PFAS concentrations among three Faroese groups, collected in 2004-2007.	11
Fig. S2. Mean serum PFAS composition (%) among three demographic groups in the Faroe Islands, 2004-07.	12
Fig. S3. The first and third principal components of Principal Component Analysis (PCA) on the serum PFAS profiles among Faroese whaling men, children and women	13
Fig. S4. Sensitivity analysis of PCA on the serum PFAS profiles among Faroese whaling men, children and women.	14
Fig. S5. Associations between exposure behavioral factors and serum PFAS levels in NHANES 2005-06 population (smooth terms only)	15
Fig. S6. Associations between exposure behavioral factors and serum PFAS levels in CHirP cohort (smooth terms only)	16

Table S1. Characteristics of NHANES 2005-06 participants included in GAM analysis (unweighted, *n*=2,120).

		Mean ± SD or N (%)
Age (years)		38.7 ± 22.1
Sex	Male	1048 (49)
	Female	1072 (51)
Race/Ethnicity		
	White	935 (44)
	Black	544 (26)
	Hispanic	565 (27)
	Other race/ethnicity or missing	76 (3)
Body Mass Index		27.0 ± 7.6
Annual household income		41982 ± 23340
Ever served in the Armed Forces of the US		
	Yes	236 (11)
	No	1884 (89)
Born in the US		
	Yes	1716 (81)
	No	404 (19)
Tap water sources		
	Do not drink tap water	378 (18)
	Community water supply	1240 (58)
	Mixed source	235 (11)
	Well	267 (13)
Floor type		
	Fully or partially covered with carpet	1956 (92)
	Smooth	164 (8)
Number of shellfish meals in past 30 days		1.6 ± 2.8
Number of finfish meals in past 30 days		2.7 ± 4.1
Have ever breastfed children		
	Yes	333 (16)
	No	1787 (84)
Have ever been pregnant		
	Yes	268 (13)
	No	1852 (87)
Had at least one menstrual period in the past 12 months		
	Yes	653 (31)
	No	1467 (69)

Table S2. Characteristics of CHirP participants included in GAM analysis (n=152).

		Mean ± SD or N (%)
Age (years)		34.1 ± 3.8
Race/Ethnicity		
	Caucasian	124 (82)
	Non caucasian	28 (18)
Annual household income ($Cdn)		
	<29,000	9 (6)
	>29,000	133 (88)
	Missing	9 (7)
Have had prior births		
	Yes	73 (48)
	No	79 (52)
Have breadfed infants before		
	Yes	70 (46)
	No	82 (54)
Microwave popcorn last year		6.6 ± 15.7
Movie theater popcorn last year		3.5 ± 6.5
Packaged foods last year		8.6 ± 20.8
Paper cups last year		138 ± 154.9
Hours spent in a vehicle per week		6.5 ± 3.4
Hours spent watching TV per week		10.0 ± 15.7
Flight time over the past 3 years		75.0 ± 59.9
Use ant bait inside home the past 3 years		0.4 ± 1.4
Use waterproof spray in the past 3 years		1.3 ± 2.5
Use waxes for leather in the past 3 years		1.0 ± 4.6
Use car waxes, sprays or polishes inside the car in the past 3 years		0.8 ± 2.9
Consuming shellfish in the past year		36.74 ± 56.3
Consuming finfish in the past year		86.9 ± 77.9
Use carpet stain repellent in the past 3 years		
	Yes	5 (3)
	No	122 (80)
	Missing	25 (16)
Use stove-top Teflon cookware		
	Yes	122 (80)
	No	30 (20)
Use firefighting foam		
	Yes	11 (7)
	No	141 (93)

Table S3. Loadings of the first three principal components (PCs) after varimax rotation.

	Component 1	Component 2	Component 3
nPFOS	0.932		0.237
brPFOS	0.928	0.211	0.106
PFHxS		0.915	
PFUnDA	0.908		0.306
PFNA	0.846	0.255	0.335
PFOA	0.848	-0.206	
PFDA	0.908		0.329
PFHpS	0.768	0.416	0.23
PFDoDA	0.871		0.324
NMeFOSAA	0.104	0.646	-0.194
PFHpA	0.247	0.743	0.12
NEtFOSAA	-0.114	0.684	0.202
nFOSA	0.406		0.841
PFDS	0.461	-0.3	-0.225
brFOSA	0.28		0.884

Table S4. Screening predictors for PFASs in the CHirP study using univariate regression.

	PFOA	PFOS	PFNA	PFHxS
Microwave popcorn last year	0.06	0.02	0.70	0.10
Movie theater popcorn last year	0.01	<0.01	0.70	0.03
Pizza deliveries last year	0.71	0.91	0.81	0.03
Takeout foods last year	0.40	0.55	0.35	0.47
Packfood heated last year	0.10	0.06	0.64	0.05
Paper cups last year	0.09	0.75	0.10	0.06
Hours spent in a vehicle per week	<0.01	0.04	<0.01	0.07
Hours spent outside per week	0.84	0.39	0.95	0.55
Hours spent at home per week	0.04	0.14	0.23	0.40
Hours spent inside per week	0.30	0.44	0.24	0.58
Hours spent watching TV per week	0.03	<0.01	0.03	0.24
Hours spent on the phone per week	0.86	0.62	0.38	0.37
Flight time over the past 3 years	<0.01	<0.01	0.01	<0.01
One-way flights over the past 3 years	0.01	0.03	0.10	0.02
Have had prior births	<0.01	<0.01	<0.01	<0.01
Have breadfed infants before	<0.01	<0.01	<0.01	<0.01
Breastfed_as_baby_cleaned	0.56	0.84	0.64	0.70
Mat_age_cleaned	0.03	0.01	0.02	0.34
Education	0.39	0.54	0.90	0.75
Annual household income ($Cdn)	0.27	0.25	0.51	0.30
Race/Ethnicity	0.69	0.81	0.21	<0.01
Mattress age(years)	0.75	0.31	0.07	0.02
Stain repellent applied to the mattress	0.25	0.21	0.65	0.38
Cooking by you	0.52	0.12	0.65	0.28
Oven_selfclean_yn	0.05	0.29	0.39	0.61
Is there any carpet in your home?	0.55	0.31	0.75	0.19
% of home that is carpeted	0.74	0.74	0.80	0.78
Carpets have been cleaned in the past 3 years	0.99	0.75	0.98	0.78
Stain repellent applied to carpets in the past 3 years	0.75	0.06	0.01	0.12
Furniture has been cleaned in the past 3 years	0.88	0.85	0.19	0.74
Stain repellent applied to furniture in the past 3 years	0.34	0.99	0.49	0.64
Goretex clothing use	0.32	0.18	0.34	0.10
Fire extinguisher use	0.73	0.74	0.04	0.86
Shred-resistant dental floss use	0.54	0.52	0.90	0.35
Bite nails	0.96	0.33	0.65	0.61
Use of a non-stick or Teflon pan	0.22	0.02	0.38	0.04
Use ant bait inside homes	0.04	0.02	0.16	0.82
Use pesticides inside homes	0.36	0.36	0.77	0.64
Use waterproof spray in the past 3 years	0.07	0.65	0.45	0.19
Use air fresheners in the past 3 years	0.29	0.58	0.85	0.45

Use waxes for leather goods in the past 3 years	0.28	0.32	0.04	0.89
Use shoe polish in the past 3 years	0.52	0.95	0.53	0.90
Use anti-static sprays in the past 3 years	0.09	0.16	0.34	0.25
Use stain removers in the past 3 years	0.54	0.27	0.13	0.75
Use car waxes, or polishes inside the car in the past 3 years	0.09	0.25	0.06	0.03
Use car waxes, or polishes outside the car in the past 3 years	0.75	0.59	0.52	0.70
Consuming finfish in the past year	0.93	0.23	0.23	0.23
Consuming shellfish in the past year	0.05	0.03	<0.01	0.99
Number of years of consuming fish at least once per month since age 10	0.04	0.10	0.31	0.12

p-value <0.05 *p-value <0.10*

Table S5 Associations between exposure behavioral factors and serum PFAS levels in NHANES 2005-06 population (parametric terms only).

Percent difference in PFAS concentrations (%) *p-value*		PFOS	PFOA	PFNA	PFHxS	PFDA	N-MeFOSAA
Variables representing exposure sources							
Ever served in the Armed Forces of the US							
	No	Ref	Ref	Ref	Ref	Ref	Ref
	Yes	-9.24	-11.27	-7.24	-17.89	-5.83	-6.59
		0.076	***0.033***	*0.183*	***0.028***	*0.314*	*0.292*
Tap water sources							
	Do not drink tap water	Ref	Ref	Ref	Ref	Ref	Ref
	Community water supply	9.72	12.92	4.00	19.78	5.37	17.93
		0.018	***0.003***	*0.335*	***0.005***	*0.225*	***<0.001***
	Mixed source	-3.00	2.15	6.40	-5.46	12.51	3.35
		0.587	*0.712*	*0.285*	*0.543*	*0.055*	*0.621*
	Well	-1.36	-9.57	-10.65	-13.14	2.03	10.45
		0.805	*0.078*	*0.050*	*0.123*	*0.741*	*0.132*
Floor type							
	Smooth	Ref	Ref	Ref	Ref	Ref	Ref
	Fully or partially covered with carpet	12.24	6.39	9.52	37.19	8.89	17.20
		0.032	*0.263*	*0.102*	***<0.001***	*0.148*	***0.013***
Other variables							
Born in the US							
	No	Ref	Ref	Ref	Ref	Ref	Ref
	Yes	31.70	40.50	2.59	45.40	-3.69	27.68
		<0.001	***<0.001***	*0.585*	***<0.001***	*0.448*	***<0.001***
Sex							
	Male	Ref	Ref	Ref	Ref	Ref	Ref
	Female	-23.16	-15.74	-10.85	-25.68	-9.45	-11.96
		<0.001	***<0.001***	***0.015***	***<0.001***	***0.047***	***0.019***
Race/Ethnicity							
	White	Ref	Ref	Ref	Ref	Ref	Ref
	Black	9.88	-16.67	9.73	-11.87	17.39	1.12
		0.015	***<0.001***	***0.020***	***0.046***	***<0.001***	*0.807*
	Hispanic	-16.41	-18.41	-16.66	-10.48	-9.68	-19.82
		<0.001	***<0.001***	***<0.001***	*0.132*	***0.038***	***<0.001***
	Other race/ethnicity	-8.50	-9.26	-2.91	-1.05	7.36	-5.20
		0.266	*0.235*	*0.721*	*0.936*	*0.416*	*0.573*
Breastfed children							
	No	Ref	Ref	Ref	Ref	Ref	Ref
	Yes	-4.82	-8.33	-7.27	0.84	-8.31	1.19

	0.282	*0.064*	*0.111*	*0.911*	*0.084*	*0.828*
Have ever been pregnant						
No	Ref	Ref	Ref	Ref	Ref	Ref
Yes	-4.36	-6.36	0.55	-18.70	6.64	-6.27
	0.345	*0.175*	*0.911*	***0.008***	*0.213*	*0.247*
Had at least one menstrual period in the past 12 months						
No	Ref	Ref	Ref	Ref	Ref	Ref
Yes	-13.90	-26.19	-14.47	-16.64	-7.90	0.71
	0.002	***<0.001***	***0.002***	***0.023***	*0.120*	*0.902*
Adjusted R-square	0.25	0.19	0.11	0.10	0.09	0.09

Table S6 Associations between exposure behavioral factors and serum PFAS levels in CHirP cohort (parametric terms only).

Percent difference in PFAS concentrations (%) *p-value*	PFOS	PFOA	PFNA	PFHxS
Use carpet stain repellent in the past 3 years				
No	Ref	Ref	Ref	Ref
Yes	52.87	17.36	75.13	68.50
	0.054	*0.408*	***0.001***	*0.121*
Use stove-top Teflon cookware				
No	Ref	Ref	Ref	Ref
Yes	15.57	12.93	-2.60	41.39
	0.174	*0.196*	*0.740*	***0.034***
Use firefighting foam				
No	Ref	Ref	Ref	Ref
Yes	-5.67	-4.59	-19.78	2.80
	0.730	*0.746*	*0.076*	*0.915*
Race/Ethnicity				
Caucasian	Ref	Ref	Ref	Ref
Non Caucasian	-15.68	-25.12	0.98	-38.75
	0.118	***0.003***	*0.903*	***0.005***
Have had prior pregnancy				
No	Ref	Ref	Ref	Ref
Yes	27.55	-18.43	-22.33	-39.62
	0.572	*0.597*	*0.444*	*0.449*
Have breastfed infants before				
No	Ref	Ref	Ref	Ref
Yes	-50.69	-45.95	-6.15	-14.12
	0.100	*0.111*	*0.846*	*0.819*
Adjusted R-square	0.44	0.58	0.45	0.34

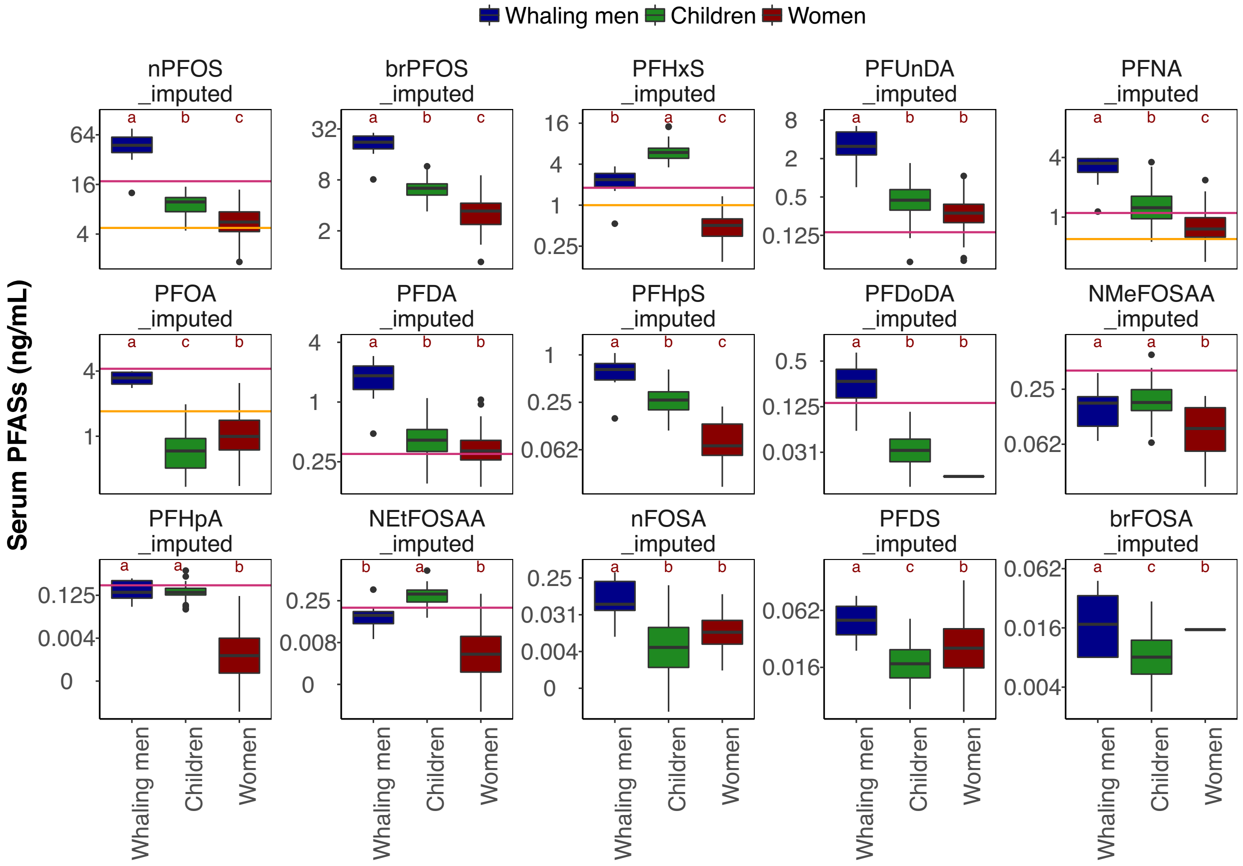

Fig. S1. Sensitivity analysis for mean serum PFAS concentrations among three Faroese groups, collected in 2004-2007. Left-censored data were imputed using the robust regression on order statistics (ROS). Red lines indicate median levels for the general U.S. population from the 2005-06 National Health and Nutrition Examination Survey (NHANES) and orange lines show median levels measured in pregnant women that participated in the Chemicals, Health and Pregnancy (CHirP) cohort in Vancouver, British Columbia, Canada in 2007-08. Means with the same letter are not significantly different (Tukey HSD test, $\alpha = 0.05$). PFOS and FOSA for NHANES and CHirP are plotted with linear (nPFOS and nFOSA) isomer measurements for the Faroese data because isomer-specific data are unavailable.

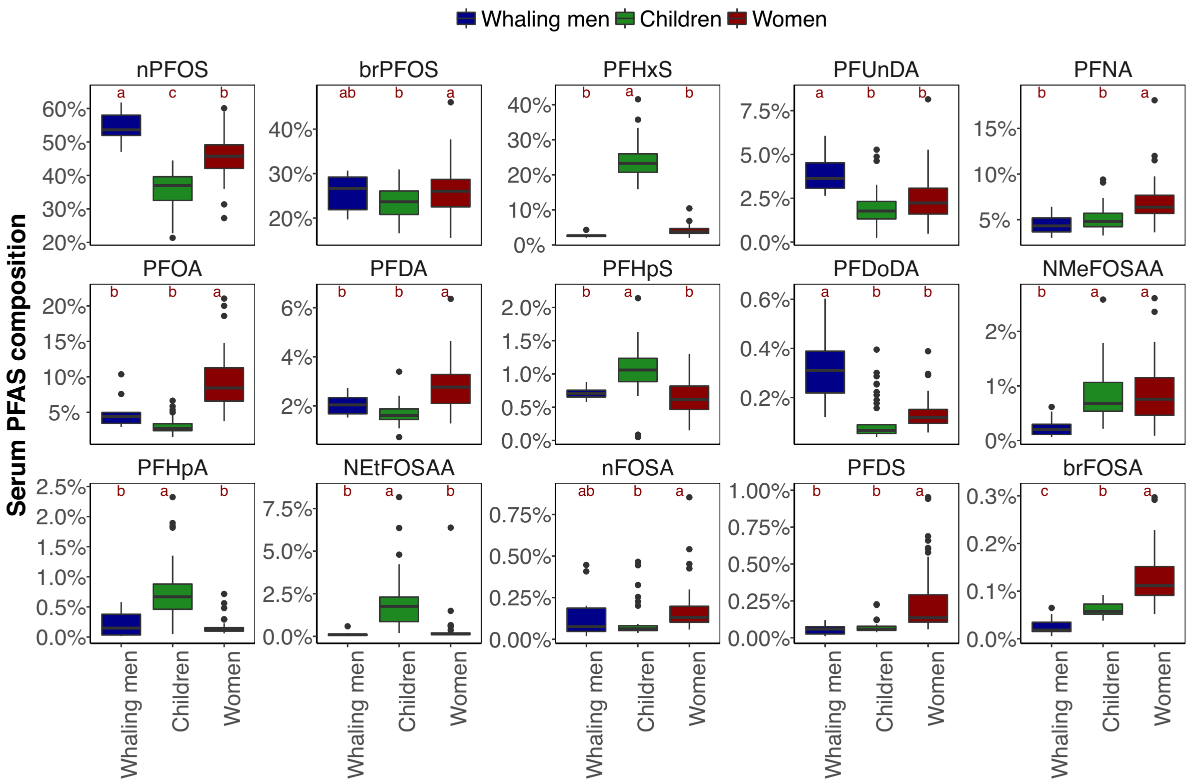

Fig. S2. Mean serum PFAS composition (%) among three demographic groups in the Faroe Islands, 2004-07. Means with the same letter are not significantly different (Tukey HSD test, α = 0.05).

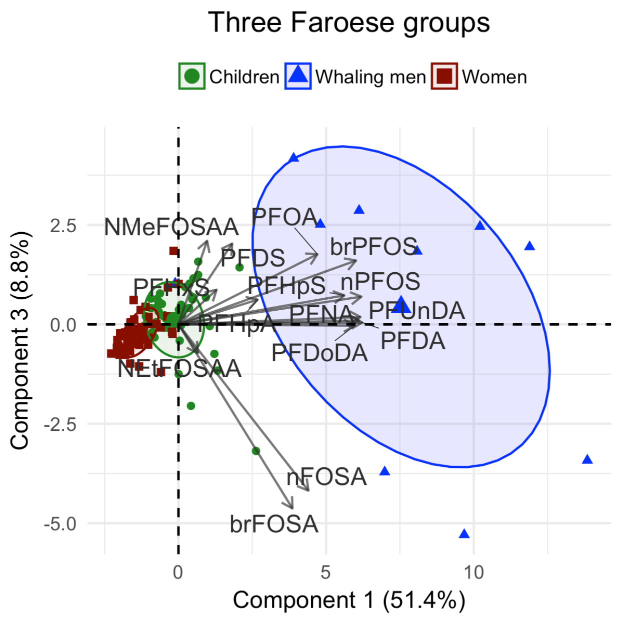

Fig. S3. The first and third principal components of Principal Component Analysis (PCA) on the serum PFAS profiles among Faroese whaling men, children and women.

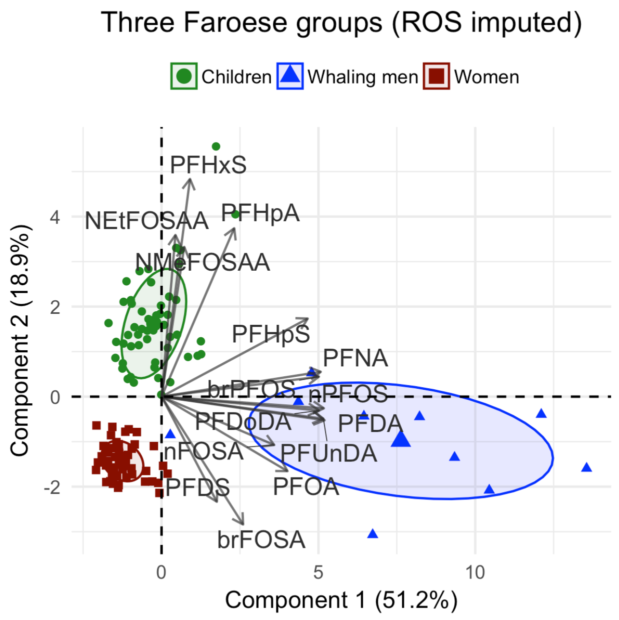

Fig. S4. Sensitivity analysis of PCA on the serum PFAS profiles among Faroese whaling men, children and women. Left-censored data were imputed using the robust regression on order statistics (ROS).

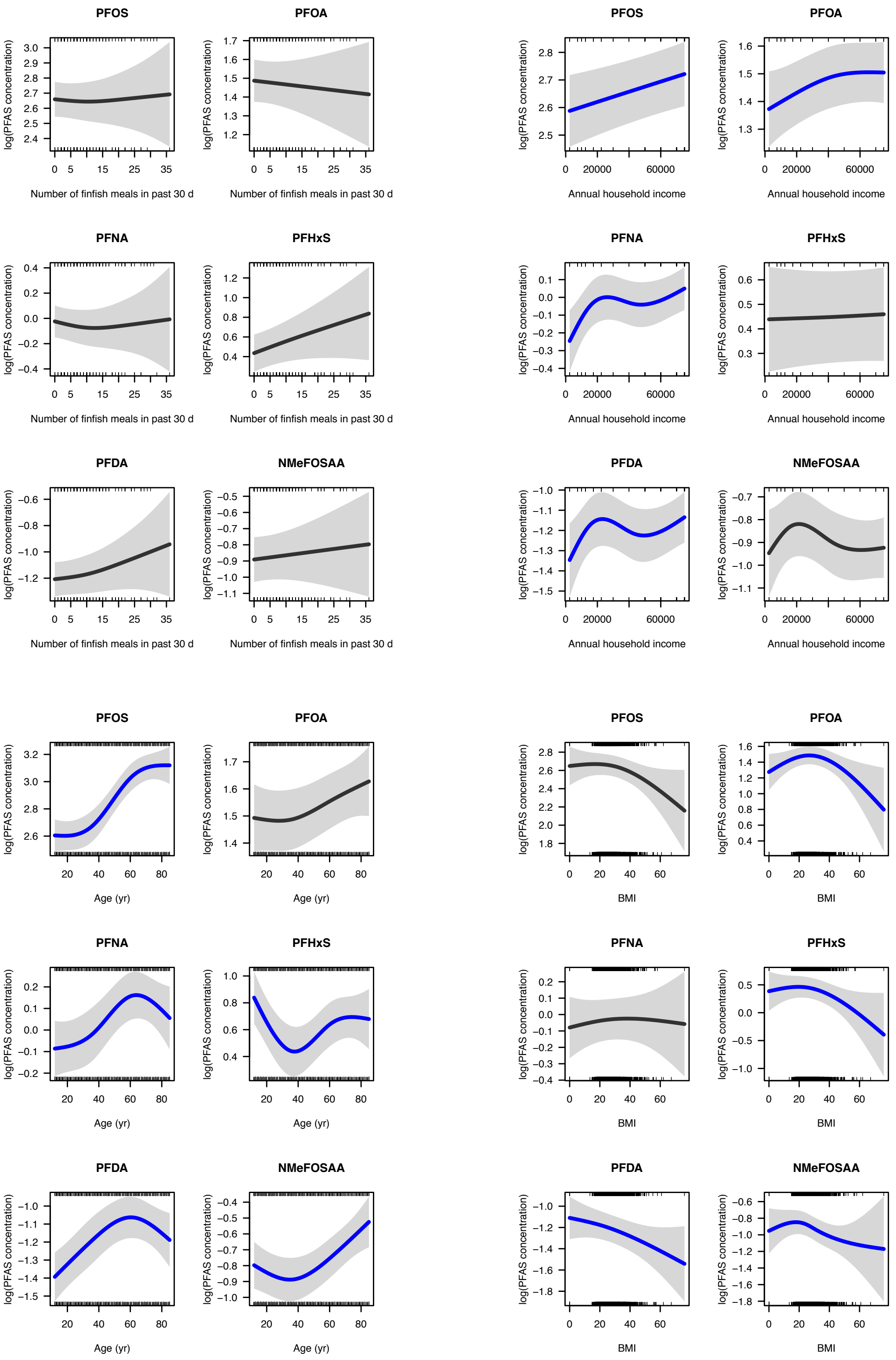

Fig. S5. Associations between exposure behavioral factors and serum PFAS levels in NHANES 2005-06 population (smooth terms only)

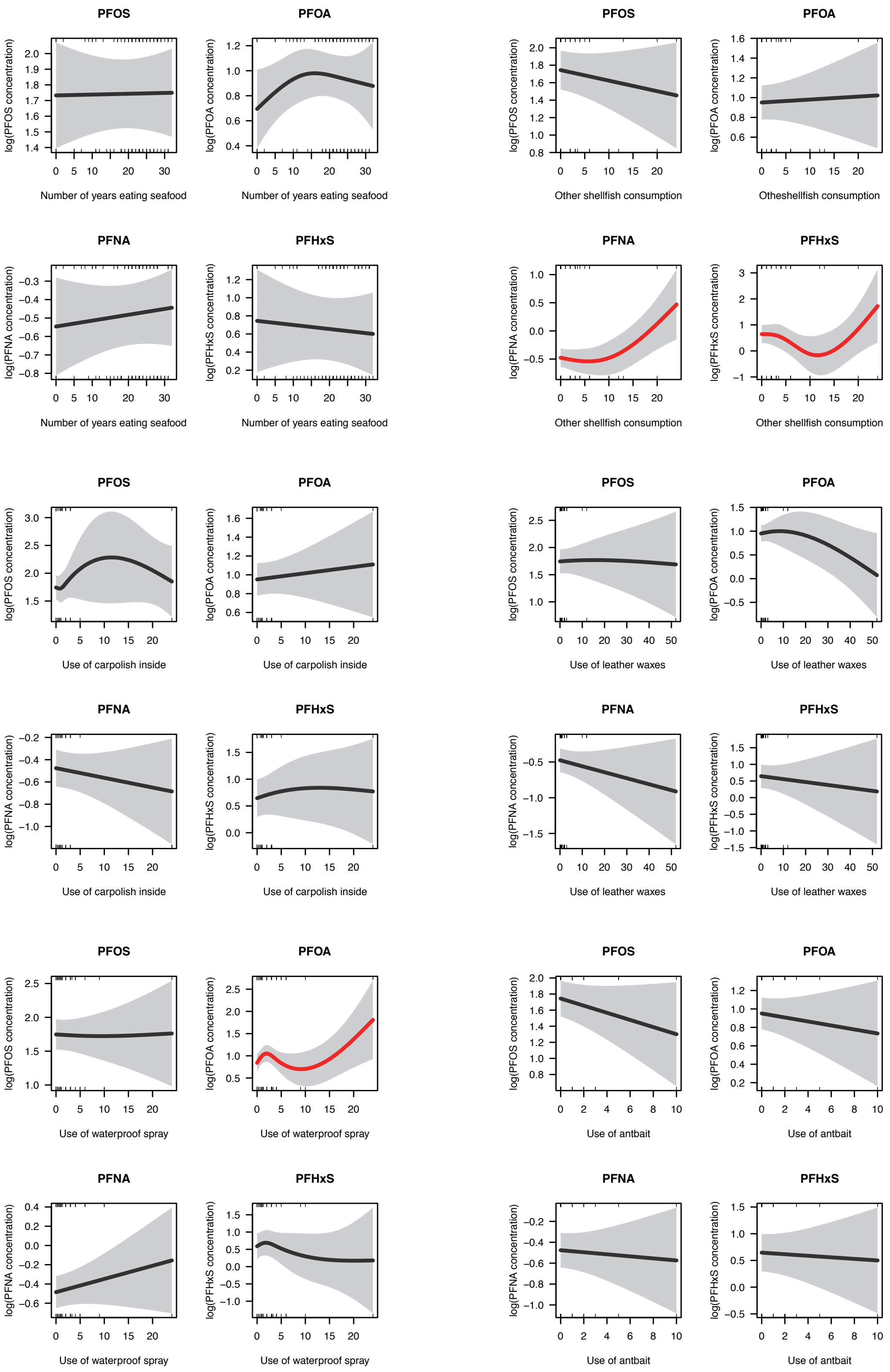

Fig. S6. Associations between exposure behavioral factors and serum PFAS levels in CHirP cohort (smooth terms only)

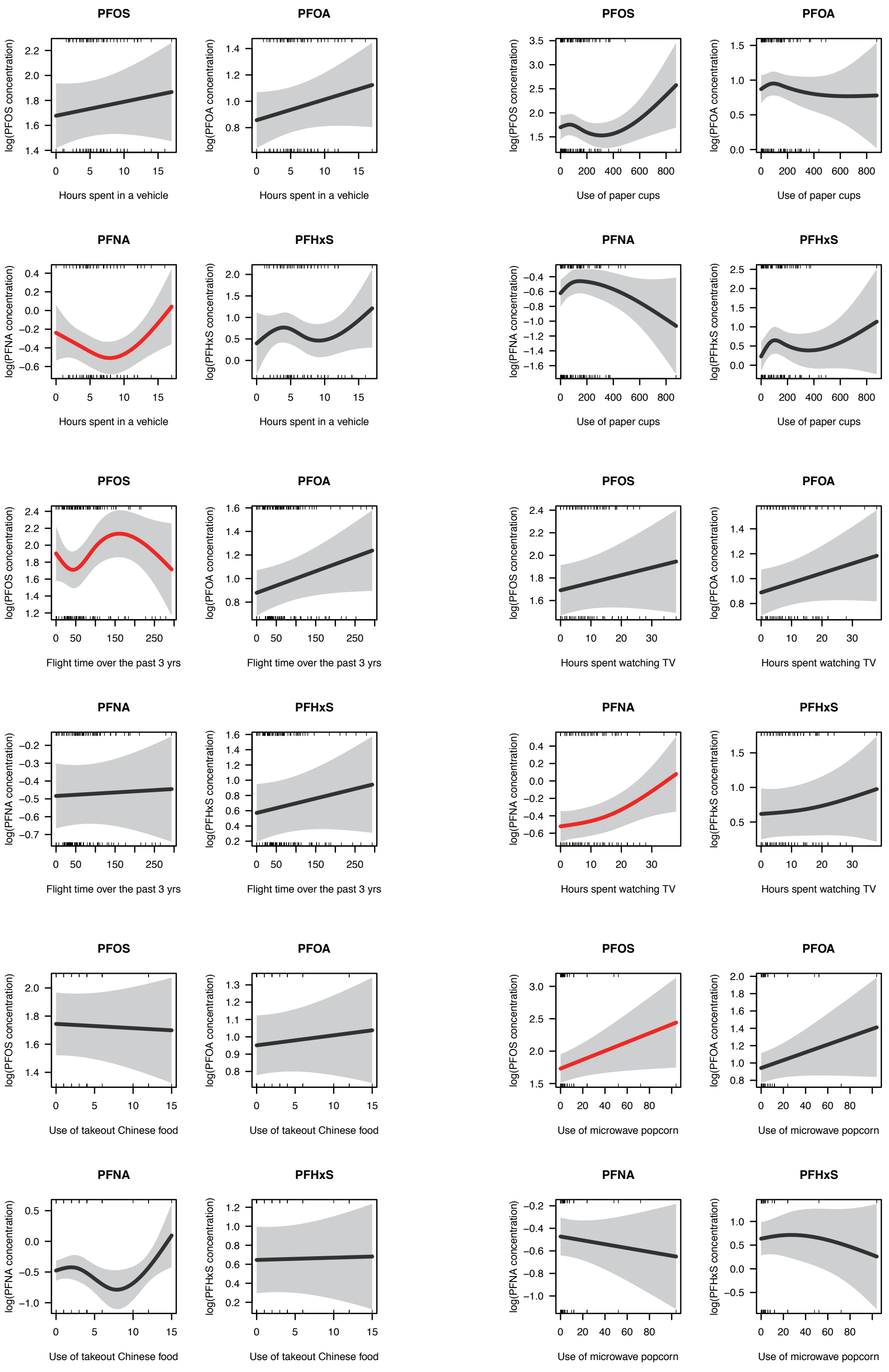

Fig. S6 (cont). Associations between exposure behavioral factors and serum PFAS levels in CHirP cohort (smooth terms only)
